# Egress and invasion machinery of malaria: an in-depth look into the structural and functional features of the flap dynamics of plasmepsin IX and X†

**DOI:** 10.1039/c8ra04360d

**Published:** 2018-06-13

**Authors:** Geraldene Munsamy, Pritika Ramharack, Mahmoud E. S. Soliman

**Affiliations:** Molecular Bio-computation and Drug Design Laboratory, School of Health Sciences, University of KwaZulu-Natal Westville Campus Durban 4001 South Africa soliman@ukzn.ac.za +27 (0) 31 260 7872 +27 (0) 31 260 8048

## Abstract

Plasmepsins, a family of aspartic proteases expressed by *Plasmodium falciparum* parasite, have been identified as key mediators in the onset of lethal malaria. Precedence has been placed on this family of enzymes due their essential role in the virulence of the parasite, thus highlighting their importance as novel drug targets. A previously published study by our group proposed a set of parameters used to define the flap motion of aspartic proteases. These parameters were used in the study of Plm I–V and focused on the flap flexibility as well as structural dynamics. Recent studies have highlighted the essential role played by Plm IX and X in egress and invasion of the malarial parasite. This study aims to close the gap on the latter family, investigating the flap dynamics of Plms IX and X. Molecular dynamics simulations demonstrated an “open and close” mechanism at the region of the catalytic site. Further computation of the dihedral angles at the catalytic region revealed tractability at both the flap tip and flexible loop. This structural versatility enhances the interaction of variant ligand sizes, in comparison to other Plm family members. The results obtained from this study signify the essential role of structural flap dynamics and its resultant effect on the binding landscapes of Plm IX and X. We believe that this unique structural mechanism may be integrated in the design and development of effective anti-malarial drugs.

## Abstract 2

A molecular dynamic study of the infiltrating machinery of malaria, an in-depth look in the flap and loop dynamics of Plm IX and X.

## Graphical abstract

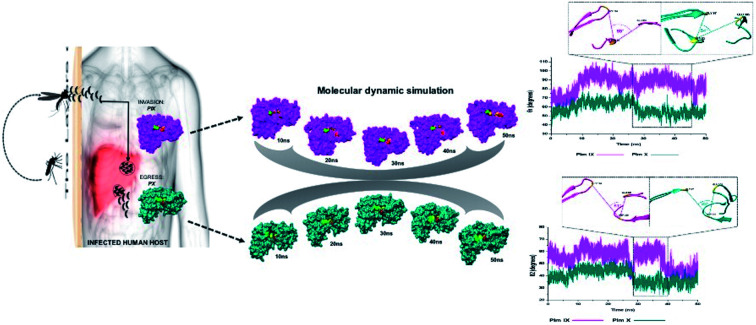

## Introduction

According to the World Health Organization (WHO), by 2016, there were an estimated 216 million reported cases of malaria worldwide.^1^ Most cases of malaria and deaths occur in Sub-Saharan Africa,^2,3^ however, regions of South-East Asia, Eastern Mediterranean, Western Pacific, and the Americas are also at critical risk.^4^ Since then, the infection rate of malaria has almost doubled, with an estimated 500 million people being infected annually, with the majority of these emerging cases being children under the age of five.^5^

In humans, the parasite is transmitted *via* the female Anopheles mosquito vector. Humans are infected by a range of *Plasmodium* species,^6^ however, the most severe and common forms of malaria are caused by *Plasmodium falciparum* and *Plasmodium vivax*.^7^*P. falciparum*, predominating in Africa, is considered to be the most virulent species being responsible for 85% of human death.^8^

The complex life cycle of *P. falciparum* embodies multiple cycles of invasion, multiplication and egress in the human red blood cells.^9,10^ Evidently, targeting the egress/invasion machinery, at its most vulnerable stage of the life cycle of the parasite may not only reduce the severity of the malarial disease, but may jointly eradicate disease transmission.^11^ Even though the process of invasion occurs rapidly, it is the only time during the parasite's life cycle when it is directly exposed to the host immune system.^12^ There are several enzymes in *P. falciparum*, that have been implicated in hemoglobin proteolysis, parasite nutrition and development, these enzyme families include; cysteine proteases; aspartic proteases; metalloproteases and dipeptidyl aminopeptidases.^13,14^

Aspartic proteases, or plasmepsins, have been identified as key mediators of cellular processes, including hemoglobin degradation for the export of *Plasmodium* proteins that essential for parasite growth/survival^15^ and particularly malarial egress and invasion.^16^*P. falciparum* possess a repertoire of 10 aspartic proteases (Plm I to X).^17^ Of the 10 Plms identified in *P. falciparum*, only Plm I, II, HAP, IV and V have been studied extensively.^18^

**Fig. 1:**
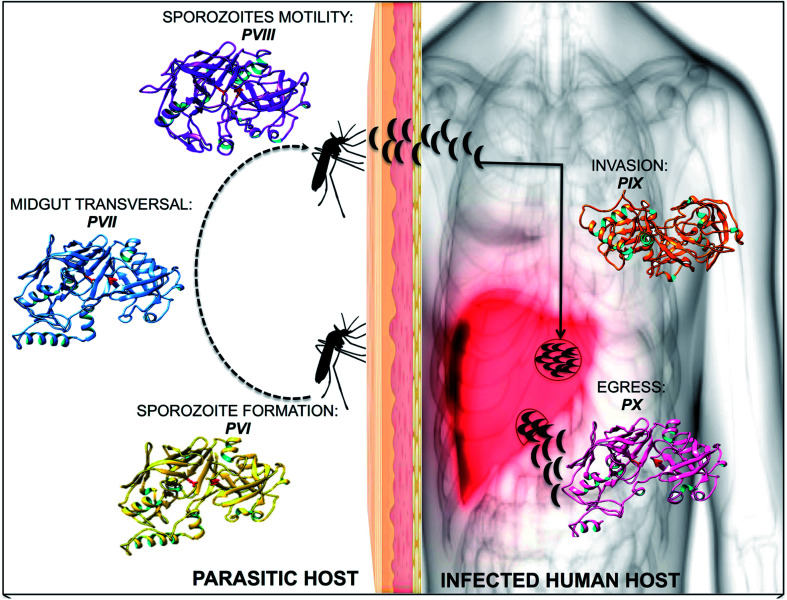
A diagrammatic representation of plasmpesin VI–X, highlighting the role of each plasmepsin towards malarial infection and invasion.

Plm VI–VIII are expressed in the vector during the parasite's intra-erythrocytic stages of sporozoite formation, motility as well midgut transversal^19^ (Fig. 1). Studies have suggested that due to their expression during the sporogonial cycle in the mosquito, these Plm's may not be ideal drug target candidates.^20^ However, understanding the molecular mechanism by which the ookinete invades the mosquito vector can form a potential approach to developing malaria transmission-blocking strategies, which may pave the way for future studies. Plm IX and X are expressed concurrently with Plm I–IV, but are not transported to the food vacuole, and have been identified as vital mediators in the progression of infection within the human host.^21,22^ The essential roles undertaken by Plm IX and X further exemplifies their choice in our study in understanding the structural and functional anomalies associated with these enzymes. The ambiguity surrounding the functional and structural features of Plm IX and X may be attributed to the absence of tertiary structures of these neglected proteases. Gaining insight into the functional and structural features of Plm IX and X may aid in the design of effective therapeutics against malaria.

The flap and flexible region of motion as well as the characteristic “twisting^23^” motion during the opening and closing of Plm's is essential in understanding the ligand binding landscape as well as conformational flexibility of Plm's. Flaps covering the active site have a dual role in Plm function: (i) structural, as interactions formed between a ligand and the flaps stabilize the ligand–protease complex, and (ii) kinetic, as flap closing induces ligand binding and flap opening induces ligand release.^24^

Previous work reported by our research group proposed parameters to measure the flap dynamics of Plm I–V,^23^ which we have implemented in this study. In the absence of crystal structures of Plm IX–X, *in silico* models were generated for Plm IX–X and further refinement of the generated models were performed using molecular dynamic simulations.

Two recent studies by Paco Pino *et al.* 2017 (ref. 25) and Armiyaw S. Nasamua *et al.* 2017 (ref. 22) have shown the expression of Plm IX and Plm X in late schizont/merozoites, and their potential involvement in egress-invasion. These studies have identified Plm IX and X as being essential for parasitic invasion and egress, and are evidently expressed in mature blood-stage schizonts and invasive merozites and fulfil indispensable but unknown functions. A conditional knockout study performed on Plm IX has revealed its role as a maturase during merozoite formation, and further substantiates it's importance for RBC invasion.^26^ Plm IX and X have been identified as crucial mediators of invasion and egress of the malarial parasite. Therefore, qualifying as promising dual targets toward malaria eradication.

This study aims in generating a better understanding of the structural and mechanistic features of Plm IX and X, considering the importance surrounding the role of flap dynamics which is a characteristic and distinguishable feature of the family of aspartic proteases as well as utilise a range of computational techniques to provide comprehensive structural and functional data that will aid in the identification or design of inhibitors specific to Plm IX and X.

## Computational methodology

### *In silico* modelling

Crystal structures generated from experimental methods, such as X-ray crystallography and or NMR analysis, have aided researchers in the understanding of the form and function of protein targets over the past decade and are continuously being proven to be useful in the discovery of small molecule based protein inhibitors.^27,28^ However, limitations such obtaining a large quantity of absolute pure protein, as well as large molecular weight are challenges often associated with the crystallization of a protein, not to mention the substantial financial investments in equipment and human infrastructure that is required. A more cost efficient and sought after approach, for the determination of the tertiary structure of many proteins, not reported in the Protein Data Bank (PDB) is *in silico* modelling.^27,29^

The amino acid sequence for *Plasmodium falciparum* aspartic proteases IX–X was accessed from NCBI,^30^ followed by the generation of the predictive 3-D structure using the Protein Homology/analogY Recognition Engine (PHYRE2)^31^ server, which selects templates for structure prediction based on secondary structural alignments. Plm's IX–X were modelled against the PDB structure of cathepsin D (4OBZ), the most closely related human aspartic protease.^19^ The sequence similarity and *Z*-score of each model to reference template 4OBZ is presented in Table S1.† A Ramachandran plot for the analyses of bond angles and torsional strain was generated using RAMPAGE^32^ (Fig. S1†). Results showed that all models generated had >90.0% of all residues in the favoured. With only a list of 12 outliers, none of which formed part of the active site of the protein. Each model was further validated using ProSA-web^33^ presented in Fig. S2.†

### System preparation

Plm IX and X were subjected to molecular dynamic simulations. The enzymes were prepared and visualised using chimera. In total, two systems were subjected to continuous molecular dynamic simulations as described below in section “Molecular dynamics and post dynamics analyses.” To verify and validate the results from the single continuous MD approach adopted herein, we ran multiple MD simulations (using three replicas) for one system and compared the results.

### Molecular dynamics and post dynamic analysis

To investigate the flap dynamics of apo Plm IX and X, a continuous 50 ns MD approach was utilised in the present study. All-atom, explicit solvation unrestrained molecular dynamic simulations were performed using the GPU version of the SANDER^34^ engine incorporated with the Amber 14 package which integrates the standard AMBER (FF14SB)^35^ force field used to describe the protein systems.

Protein systems were modelled using the standard AMBER (FF14SB)^36^ force field present in the Amber 14 package. To generate topologies of the protein systems and to ensure neutralization of the respective proteins system prior to the production run, the addition of hydrogen atoms and counterion were implemented by the LEaP module of Amber 14. The protein system was then immersed in orthorhombic TIP3P^35^ water box to model the water molecules explicitly, at a distance of 10 Å from all protein atoms. The particle mesh Ewald (PME) method^37^ in the Amber 14 package was incorporated to compute the long-range electrostatics interactions in the molecular dynamic simulation with a van der Waals cut of distance of 10 Å. Systems were subjected to consecutive minimization steps, the initial partial minimization followed by full minimization.

An initial minimization of 2000 steps was carried out with an applied restraint potential of 500 kcal mol^−1^. This was followed by a full minimization of 1000 steps carried out by conjugate gradient algorithm without any restraints. All systems were gradually heated from 0 to 300 K for 50 ps in the canonical ensembles (NVT), by the application of harmonic restraints of 10 kcal mol^−1^ Å and collision frequency of 1.0 ps^−1^ to all solutes in the system. The simulation temperature was controlled using the Langevin thermostat. All systems were equilibrated at 300 K in an NPT ensemble for 500 ps with no restraints and a constant pressure (1 bar) was maintained using the Berendsen thermostat. The SHAKE algorithm was used to constrict the bonds of all hydrogen atoms. Continuous MD was performed on all systems for 50 ns in an NPT ensemble with a constant pressure of 1 bar, constant temperature of 300 K and a pressure coupling constant of 2 ps.

Trajectories were saved every 1 ps and further analysed (for example, RMSD, RMSF, distance (*d*_1_) and dihedral angle *Φ*) using the CPPTRAJ^38^ module incorporated Amber 14. As an additional validation of the continuous MD approach utilised in the current study, a multiple MD approach was performed for one system. In which three 50 ns MD runs with different initial velocities were performed and the average trajectory analysed. The trends for both approaches are similar, confirming that the reported approach is reliable and valid. The graphical interface of UCSF chimera^39^ was used to visualise all enzyme structures, and data was analysed and plotted using the GUI of Microcal Origin data analysis software version 6.5.^29^

### Parameters used in this study

**Fig. 2:**
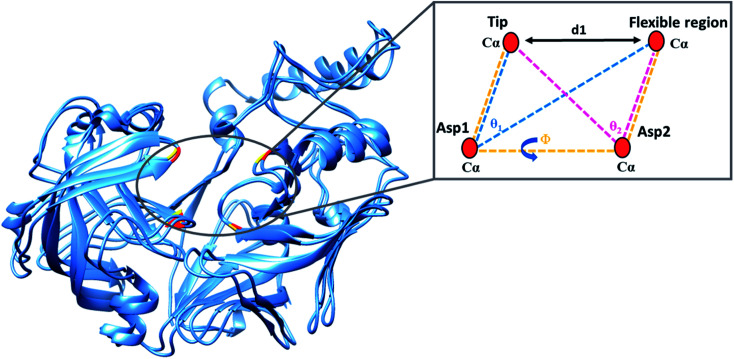
Schematic representation of the parameters used to define the flap-structure motion: *d*_1_ the distance between the flap tip and flexible region hinge residue, the dihedral angle *Φ* and the TriCα angles, *θ*_1_ and *θ*_2_. Asp1, Asp2, the flap tip and flexible region of PMIX are presented in gold with the respective regions of PMX represented in red.

The present study utilised parameters previously proposed by our group to more accurately describe flap dynamics for aspartic proteases, which focused on Plms I–V. This study is a continuation of our work but primarily of Plm IX and X. These parameters are represented schematically in Fig. 2.

## Results and discussion

### Exclusive structural features of plasmepsins

The active site of the *P. falciparum* Plm comprises of two catalytic aspartic acids, covered partially by a β-hairpin flap, a feature commonly shared with Plm IX and X.^40^ Numerous studies have shown that residues lining the active site such as the flap and flexible loop undergo large conformational changes instrumental for ligand binding. It has been reported that the flap and flexible domain of Plm I–V display a “twisting” dynamic motion which can be observed during the opening and closing of the active site region.

**Fig. 3:**
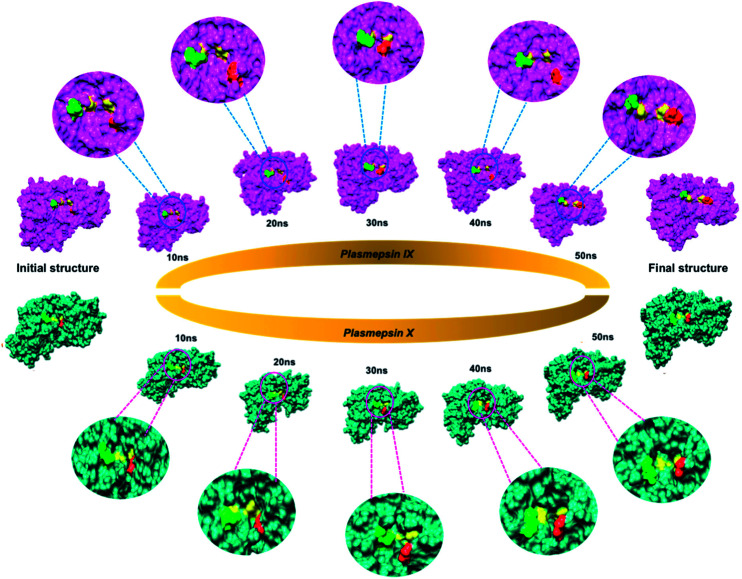
Flap and flexible loop region movements of apo Plm IX (pink) and X (blue) throughout a 50 ns molecular dynamics simulation. The catalytic aspartate residues of Plm IX and X are highlighted in yellow, the flap tip in red and hinge residue in green.

In this study, we could highlight this distinct feature shared in Plm IX and X from the visual analysis of the snapshots presented in Fig. 3 of Plm IX and X. The apo tertiary structures transition between an open and semi-open conformation throughout the simulation. Present in all orthologs of Plm's are the regions surrounding the catalytic active sites, while there is a distinctive structural difference between aspartic protease, predominantly in the loop regions at the top and bottom of the model. Plm I–X exhibit high structural similarity, as can be seen from the superimposed structures of Plm I, II, III, IV, VI, VII, IX and X (Fig. S3†), as opposed to their sequence composition and display an overall conservation of the active site (Fig. S4†). Although there is a deviation in the sequential composition of Plm's, there is an overall conservation of the position of the catalytic aspartic residues and hydrophobic residues proximal to each binding cleft of Plm IX and X to other plasmepsins.^41^

Plm's are often associated with notable substrate specificity. This specificity is linked to the sequence heterogeneity of residues lining the active site, such as the flap and flexible loop domains. To date, there are no approved inhibitors against Plm. This paves the way towards the design of novel inhibitors that are more resilient against resistance. For Plm's, *d*_1_ accurately describes the opening and closing of the flap-structure, and the dihedral angle *θ* can be used to gain insight into the twisting motion of the flap-structure.

### Mechanistic flexibility of plasmepsin IX and X

Root mean square deviation (RMSD) analysis projects information that can be used to measure the stability and disorder or stereochemical variability across a set of structural models, with the RMSD being complementary to the crystallographic B factor. Analysis of RMSD were performed to validate the stability of the apo systems of Plm IX and X (Fig. S5†), and to ensure accuracy of successive post-dynamic analyses. The potential energy (PE) was also computed across the 50 ns MD simulation of Plm IX and X to further validate the convergence of each system. From the PE plots presented in Fig. S6,† the convergence of both systems was achieved within the 50 ns MD trajectory, which corresponds with the stability attained at 30 ns of the RMSD trajectory of Plm IX and X. The average PE for Plm X was substantially higher than that of Plm IX which were −158461.37 and −163213.16 respectively. The average PE correlate with the average RMSF values for both system, further accentuating a greater degree of stability in Plm IX as opposed to Plm X. This finding coincides with the “twisting motion” being more prominently exhibited by IX as opposed to X.

The residues used to calculate the distance (*d*_1_), dihedral angle (*Φ*), TriCα *θ*_1_ and *θ*_2_ can be found in Table 1.

**Table 1:** Residues used to calculate the distance (*d*_1_), dihedral (*Φ*), TriCα *θ*_1_ and *θ*_2_

	Plasmepsin IX	Plasmepsin X
Distance (*d*_1_)	Gly124-Glu331	Gly97-Glu305
Dihedral (*Φ*)	Gly124-Asp80-Asp259-Glu331	Gly97-Asp53-Asp232-Glu305
TriCα *θ*_1_	Gly124-Asp80-Glu331	Gly97-Asp53-Glu305
TriCα *θ*_2_	Glu331-Asp259-Gly124	Glu305-Asp232-Gly97

### Plasmepsin residue fluctuations

Structural comparison of models Plm IX and X to well characterized aspartic proteases, observed no structural abnormalities. For quantifying the flexibility, Root Mean Square Fluctuation (RMSF) calculations were utilized.

Although both models displayed erratic behaviour in comparison to Plm I–V, Plm IX and X display a similar trend in the fluctuation observed in key residues. The average fluctuation of the Plm IX and X were relatively higher than the fluctuation observed in the key residues, (Table 2). Displaying lower fluctuation of key residues in relation to the average fluctuation observed in both systems.

**Table 2:** RMSF values of apo Plm IX and X for the binding site residues, tip and hinge residue, and total average fluctuation

	PLASMEPSIN IX	PLASMEPSIN X
ASP 1	0.6178	0.7249
ASP 2	0.6878	0.7213
FLAP TIP	3.6074	2.4348
HINGE	1.2962	1.4321
AVERAGE	6.44	12.63

It was interesting to note that the average values of fluctuation observed in the key residues of Plm IX and X (Table 2) follow a similar trend to those observed for Plm's I–V, displaying slightly greater flexibility in the aspartic residues, flap tip and hinge region as opposed to other Plm family members.^42^ Plm IX and X displayed greater flexibility particularly in Asp 2 and the hinge residue as opposed to Asp 1 and the flap tip, which coincides with the similar observation made in Plm I–V. The increased flexibility interjected by Asp 2 and the hinge residue may be linked to the “twisting” characteristic of the opening and closing of the active site to enhance and stabilize ligand binding. Although there is a general trend of similarity shared by Plm I–V and Plm IX–X, the average fluctuation observed in Plm IX and X, was substantially higher than that observed in Plm I–V. This may be attributed to the use of homology models as opposed to crystal structures which display a greater level of stability.

In the absence of X-ray crystal structures, *in silico* tools are often implemented to assist structure based drug design and eliminate occurrences of drug resistance. Some of the setbacks associated with the formation of protein crystals are the presence of highly flexible and disordered regions, and this bottleneck cannot be easily overcome even with the use of “predictive” tools.

**Fig. 4:**
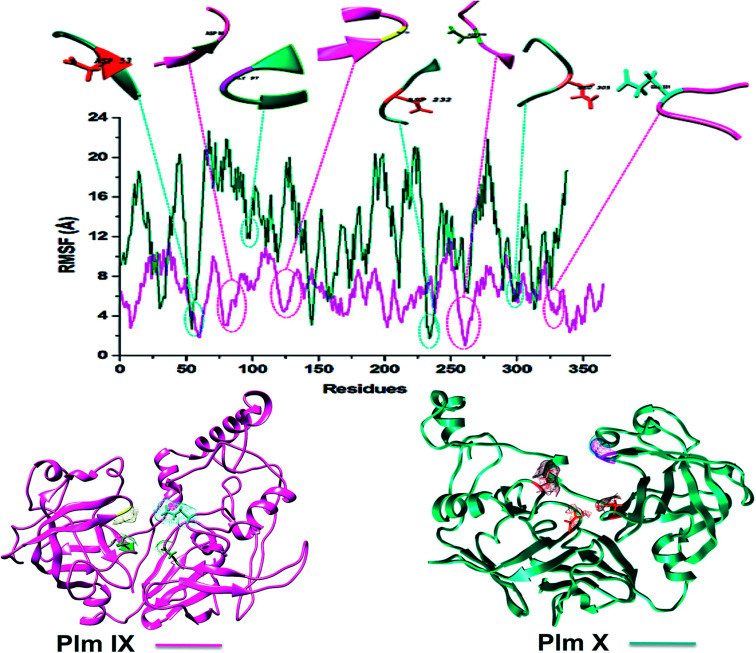
C-α RMSF plot of *Plasmodium falciparum* Plm IX (pink), X (blue), respectively.

Although Plm IX and X display a high level of structural similarity to Plm I–V, both Plm IX and X encompass highly flexible and disordered regions^19^ as can be observed in Fig. 4 and Table 2. Even in the presence of these highly flexible and disordered regions, the key residues making up the active site of Plm IX and X display levels of flexibility relatable to other Plm's, with no drastic deviation in flexibility for these regions.

Proteins destined for export contain an N-terminal signal sequence (RxLxE/Q/D) known as the *Plasmodium* export element (PEXEL).^43^*P. falciparum* Plm IX is one of the Plm that contains a PEXEL motif in its sequence (amino acid position 45–50), which marks the protein for export into the host red cell. This motif proves essential for protein export into the host red blood cell. When compared to Plm's I–VIII, *P. falciparum* Plm's V, IX and X also have comparatively large low complexity insert regions (LCR). Plm X contains 4 LCR's which is a greater quantity of LCR regions in comparison to other Plm's. The LCR contain non-random, limited alphabet amino acids. And are generally located in the solvent exposed hydrophilic loops of proteins. Although Plm IX present in all species of *Plasmodium* that contain LCR's, there is an increased number of these regions in the proteins encoded by species that infect humans.^41^ All these structural discreteness attributes to the elevated levels of fluctuation observed in both Plm IX and X as observed in Fig. 4.

### Distance [*d*_1_] and dihedral angle [*Φ*] analysis

**Fig. 5:**
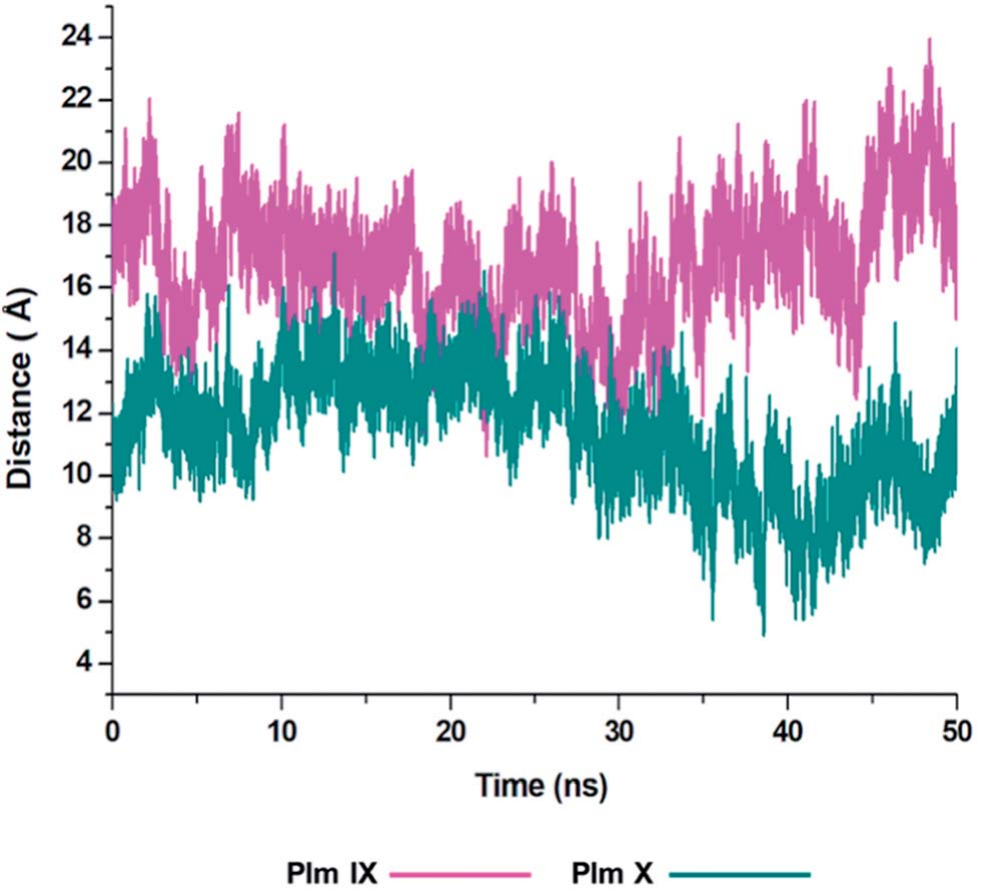
Graph showing the fluctuation in the distance, *d*_1_ as presented in Fig. 2 of Plm IX (pink) and Plm X (blue).

Although the opening and semi-closed conformation observed in Plm's IX and X, is clearly visible in Fig. 5. The distance (*d*_1_) between the flap tip and flexible loop referred to as the hinge residue is pivotal for accurately describing the opening and closing of the flap-structure, with the dihedral angle (*Φ*) being calculated to gain insight into the twisting motion of the flap structure.

From the analysis of the distance (*d*_1_) between the flap tip and hinge residue of the flexible loop we could visually see the opening and closing conformations of Plm IX and X throughout the simulation.

With Plm IX exhibiting an open conformation from 10–16 ns, 35–50 ns, curled conformation at 18–34 ns, and semi-closed conformation at 30–34 ns. Whereas, Plm X exhibiting an open formation at 12–14 ns, curled conformation at 23–28 ns and closed conformations at 5–10 ns and 39–48 ns. At 30 ns we observe a shift in the dynamics of the flap tip and hinge residue of Plm X, as these regions move closer together we observe a significant decrease in the distance between the flap tip and hinge residue. The Plm X system maintains this distance and dihedral angle, with minimal fluctuation being observed while the system moves towards a more closed conformation. The meaningful change observed in the distance are presented in Table 3, with the opening and closing motion being more clearly prominent in Plm IX as compared to Plm X.

**Table 3:** Measurement of the distance by which the flap-structure moves, measured in Angstroms

	PLASMEPSIN IX	PLASMEPSIN X
AVERAGE	27.3671	20.5052
MINIMUM	13.5465	10.8648
MAXIMUM	21.27215	16.12234
*Δ* a	13.8207	9.6404

a Change between maximum and minimum distance.

There is a large sense of ambiguity surrounding Plm IX and X, thus this study validates the flap-structure motion of an opening and closing conformation, of both enzymes as presented by other aspartic proteases. An ideal inhibitor may target the flap tip and hinge residues comprising of two glycine residues present in both enzymes and two negatively glutamate residues present in Plm IX and X respectively. Previous studies have highlighted the Gly–Gly omega bond in the glycine rich flap, which undergoes a *cis*–*trans* isomerization, at incremental time scales initiating the flap opening.^44^ The presence of these glycine residues makes the curling motion of the flap region possible. Targeting this specific domain may aid in the design of potent structure-based inhibitors against plasmepsins such as allosteric inhibitors targeting flap pockets, hindering the opening and closing of the binding cavity.

**Fig. 6:**
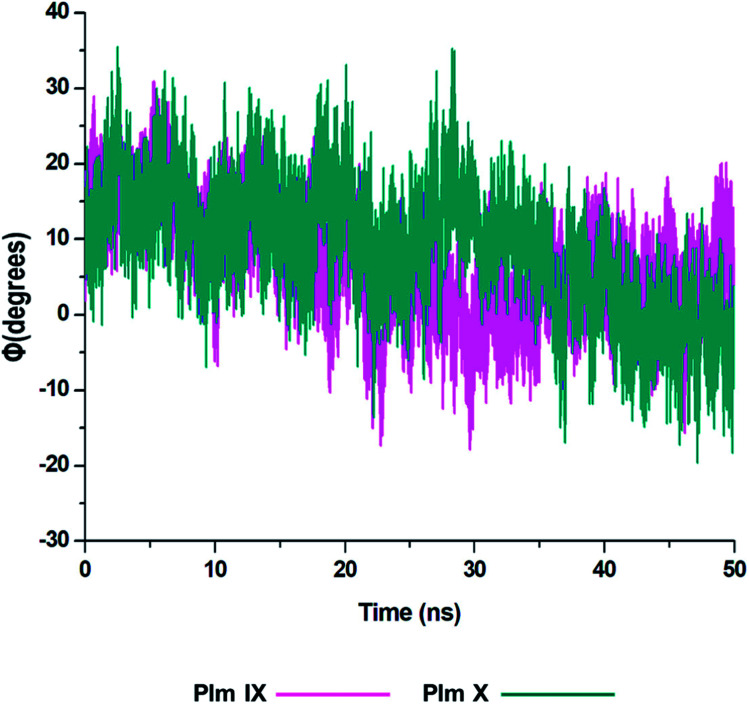
Graph of the dihedral angle throughout the 50 ns simulation, of Plm IX (pink) and X (blue).

From the analysis of the dihedral angles of Plm IX and X we can distinctly see the twisting motion observed from the graphs presented in Fig. 6 respectively. The dihedral angle *Φ*, which is a change from negative/positive and or positive/negative, we observe the twisting motion more distinctly displayed in Plm IX in comparison to Plm X which coincides with the increase in *d*_1_. At open conformations, where the distance is at its maximum which is at 42–50 ns the angle changes from −10° to 20.25°, another twist is observed at 30–35 ns the angle changes from −19° to 12.5° projecting a twisting motion of the flap region. In Plm X we observe a major twist observed between 23–28 ns, the angle transcends from 9.5° to 35°, corresponding to the curled conformation of the flap tip and hinge residue. The dihedral angle of the twisting motion is much more defined in Plm IX, as opposed to Plm X and this can also be correlated to the average distance in Plm IX, which is 21.27 Å as compared to that of Plm X, which is only 16.12 Å.

### TriCα angles *θ*_1_ and *θ*_2_

**Fig. 7:**
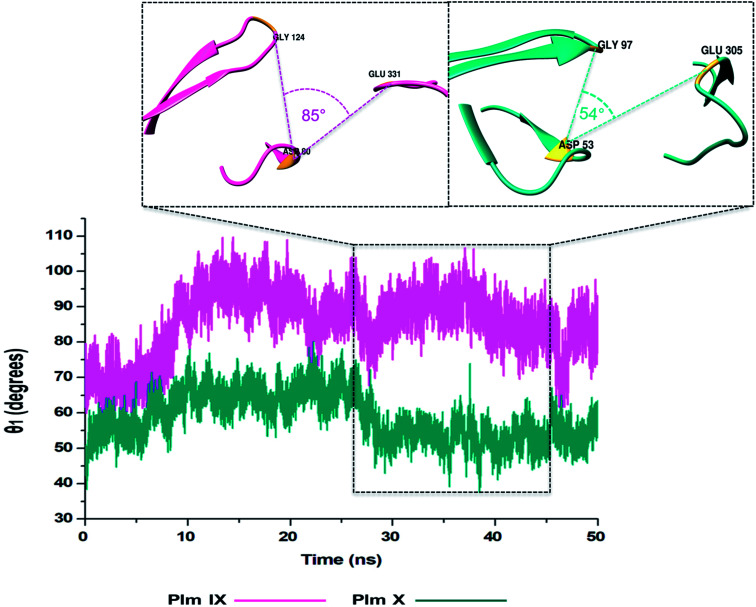
Graph showing the TriCα angles *θ*_1_ of Plm IX (pink) and X (cyan), respectively.

**Fig. 8:**
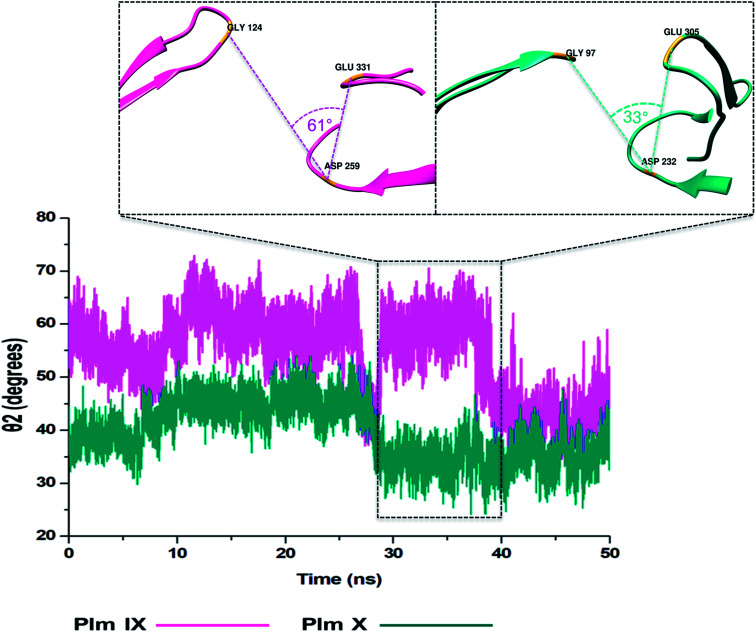
Graph showing the TriCα angles *θ*_2_ of Plm IX (pink) and X (blue), accordingly.

In this present study, we can see *θ*_1_ and *θ*_2_ follow similar trends to each other as observed in Fig. 7 and 8. The trend observed in *θ*_1_ and *θ*_2_ corresponds to the fluctuations observed in *d*_1_ for Plm IX, with the maximum *θ*_1_ and *θ*_2_ values coincide with opening of the flap structure, as the flap and flexible loop move away from each other exposing the active site. The minimum *θ*_1_ and *θ*_2_ values correlate with a more closed flap structure for Plm X as the flap folds inwards toward the active and the flexible loop recoils closing the active site.

Overall, as the flap-structure transitions into more open conformations (increase in *d*_1_), both *θ*_1_ and *θ*_2_ increase, whereas as the flap-structure moves toward a more closed conformation (decrease in *d*_1_), *θ*_1_ and *θ*_2_ decrease consequently. However, there is distinctive observation in Plm IX, there is an observed decrease in *θ*_2_ as opposed to that of *θ*_1_ between 40–50 ns, where the system maintains an open conformation. This decrease correlates to the conformational change which may be due to “closure-inducing” ligand binding. The selected residues making up *θ*_2_ may actively aid in stabilization of the ligand once it enters the catalytic site in the open conformation. This unique further illustrates the structural versatility of Plm IX and its importance in drug design. Similarly, a very distinct observation can be made for Plm X as we observe an increase in both the *θ*_1_ and *θ*_2_ angles from 10–30 ns, where the flap and hinge region remain in distal proximity, but in a compact conformation covering the aspartic catalytic domain. At 30 ns, there is an abrupt decline in the angles from 50° to 27°, where the flap tip and hinge region are near each other observed throughout the 50 ns simulation and reaches a state of stabilization.

### Radius of gyration (ROG)

**Fig. 9:**
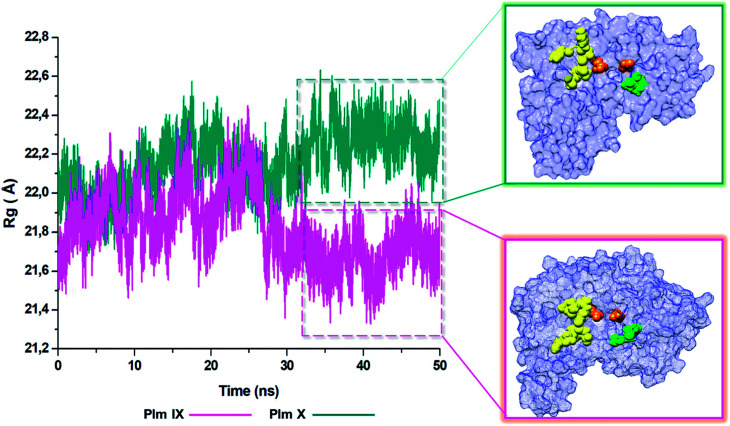
Radius of gyration (ROG) of Plm IX (pink) and Plm X (blue).

The radius of gyration is indicative to the compactness of the tertiary structure of a protein that is, how folded or unfolded a protein is, and gives insight into the stability of biological molecules during the MD simulation.^45^ The Rg for Plm IX and X, were investigated in this study, displaying a similar trend as observed for that of *d*_1_ and *θ*_2_. The Rg of Plm IX exhibited fluctuation, as the flap and flexible region moved between an open and closed conformation. The increase in the fluctuation observed from 40 ns, of the Plm IX is where the system reaches a state of semi-stabilization in an open conformation. We can see from Fig. 9 at 40 ns the flap tip and hinge residue move further apart, and maintains an open conformation at 50 ns, which coincides with an increase in the distance between the flap tip and flexible loop. The observations made for Plm X is like the observations made for *d*_1_ and *θ*_2_, we see a decrease in the Rg at 30 ns where the flap and flexibles residues are at its closest distance to each other. After 30 ns the system experiences gradual fluctuation as the flap-structure remains in a semi-closed conformation.

## Conclusions

Only a handful of proteases have been discovered and characterized prior to the completion of genome sequencing for *P. falciparum*. There are currently 6301 published crystal structures of aspartic proteases from experimental data (X-ray/NMR analysis),^46^ amidst there are still no crystal structures of Plm IX and X. This study aimed to pave the way towards the design of potent inhibitors of Plm IX and X.

It is not coincidental that glycine mediates the role of the flap tip, observed in both Plm IX and X, as the amino acid glycine possesses enhanced conformational flexibility upon folding to form part of the α-helix.^47^ Flexibility and mobility of the flap tip is essential, as it lies perpendicular over the catalytic site, mediating the binding of inhibitors and or substrates. The presence of the glycine rich flap of Plm IX (Phe-Gly-Thr-Gly) and Plm X (Phe-Gly-Ser-Gly) are highly mobile and flexible and transitions between semi-open and open conformations in the absence of a ligand. The role undertaken by the flap tip regarding the opening and closing of the catalytic domain remains key in generating the twisting motion.

The hinge residue presents in the flexible loop of Plm IX and X, are represented by glutamate. Glutamate carries a hydrophilic acidic group with a strong negative charge. The presence of this negatively charged amino acid positioned towards the outer surface of a protein promotes water solubility and stability of a protein. This validates the reduced fluctuation observed in the hinge residue of Plm IX and X as compared to that of the flap tip. Information extracted from the TriCα angles and *θ*_2_, we observe an increase in fluctuation during the open conformation of the models of Plm X, and a decrease in Plm IX. This decrease may aid in the effective binding a ligand during the open conformation, further implementing stability. This fluctuation may be stabilized primarily in the flap and flexible regions when bound to an inhibitor as observed in Plm II.^48^

From the average distance of Plm IX and X being greater than 15 Å respectively, illustrates the flexibility and ability to accommodate inhibitors of variable size.^17^ This flexibility is governed by the N-terminal flap and flexible loop. As Plm IX and X are structurally similar we do observe a trend in the transition between the open and closed conformation, however the transition between these two states of Plm IX and X are experienced at independent time intervals. Thus, displaying the activity of these aspartic proteases are not predictable in their conformational flexibility and dynamic behaviour. Even though the overall RMSF fluctuation of Plm X is greater than Plm IX, there is greater fluctuation in the flap tip of Plm IX than Plm X of 3.6074 Å and 2.4348 Å accordingly which corresponds with the twisting motion which is more prominently exhibited by Plm IX than X.

The results obtained in the present study, have signified the essential role undertaken by the flap dynamics and provides new insight into the relevant conformational changes of the flexibility of Plm IX and X. Which will ultimately aid in the design and development of novel anti-malarial drugs.

## Conflicts of interest

There are no conflicts to declare.

## Supplementary Material

RA-008-C8RA04360D-s001
