# Supplementary material for: Egress and invasion machinery of malaria: an in-depth look into the structural and functional features of the flap dynamics of plasmepsin IX and X
Source: RSC Adv. 2018 Jun 13;8(39):21829–40. doi: 10.1039/c8ra04360d (PMC9081207; doi:10.1039/c8ra04360d)
Supplement: RA-008-C8RA04360D-s001 [file RA-008-C8RA04360D-s001.pdf]

Electronic Supplementary Material (ESI) for RSC Advances.
This journal is © The Royal Society of Chemistry 2018

Egress and Invasion Machinery of Malaria: An In-depth Look into The Structural and Functional Features of The Flap Dynamics of Plasmepsin IX and X

Supplementary Material

Geraldene Munsamy[a], Pritika Ramharack and Mahmoud E. S. Soliman[a*]

[a] Molecular Bio-computation and Drug Design Laboratory, School of Health Sciences, University of KwaZulu-Natal, Westville Campus, Durban 4001, South Africa

*Corresponding Author: Mahmoud E.S. Soliman

Email: soliman@ukzn.ac.za

Telephone: +27 (0) 31 260 8048, Fax: +27 (0) 31 260 7872

PLASMEPSIN	*TEMPLATE*	*SEQUENCE IDENTITY*	*Z-SCORE*
**IX**	4OBZ	35%	-6.16
**X**	4OBZ	35%	-6.22

Table S1. The Z-score and sequence identity of 4OBZ template used for Plm IX-X

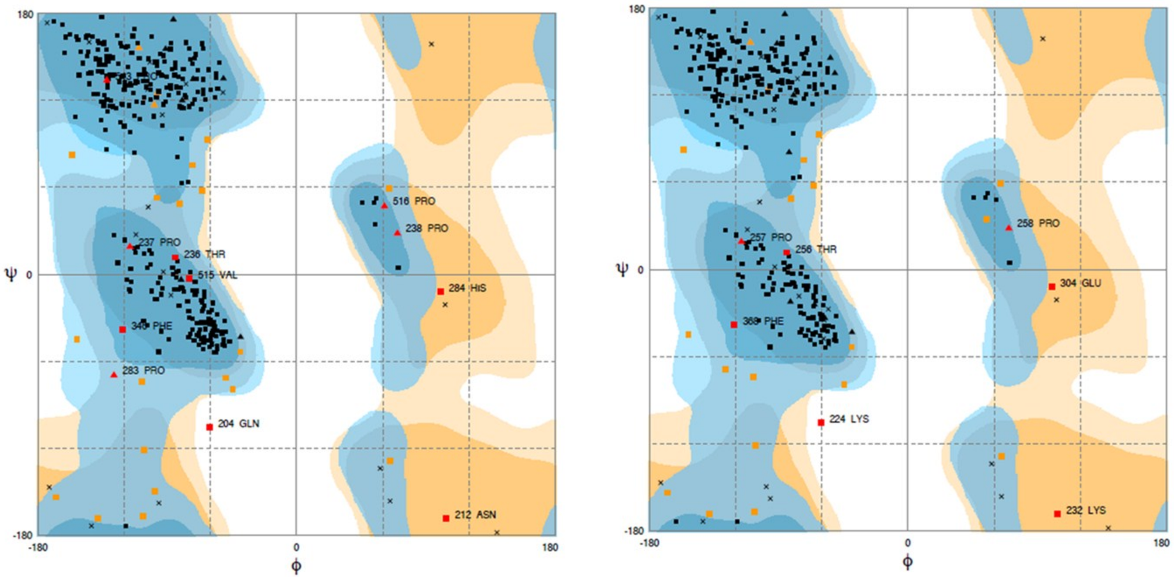

Figure S1. Ramachandran plots for models Plm IX (left) and Plm X (right).

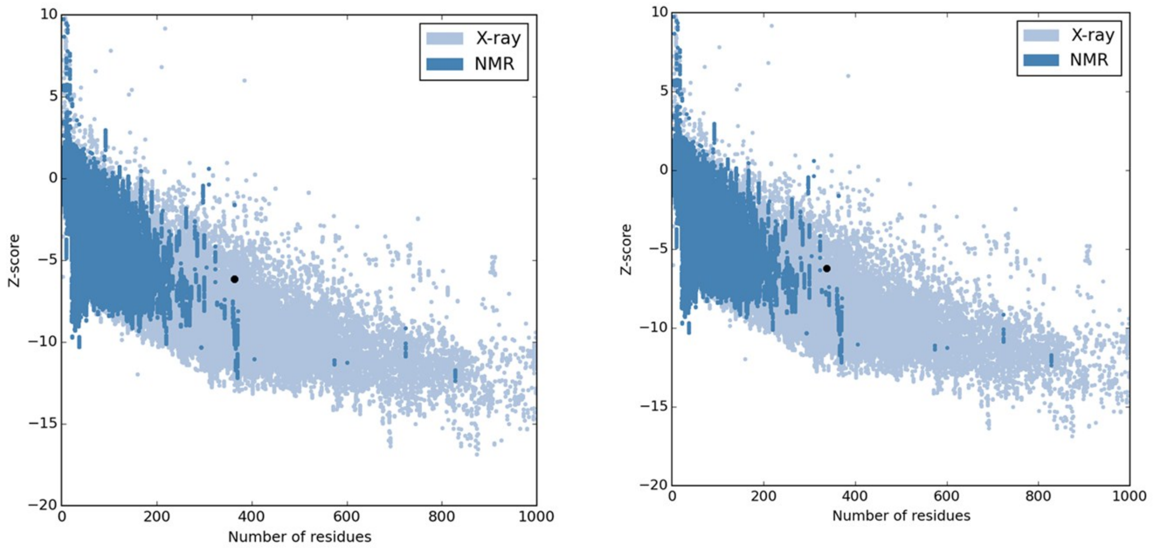

Figure S2. Overall model quality generated using ProSA-web of Plm IX (left) and Plm X (right).

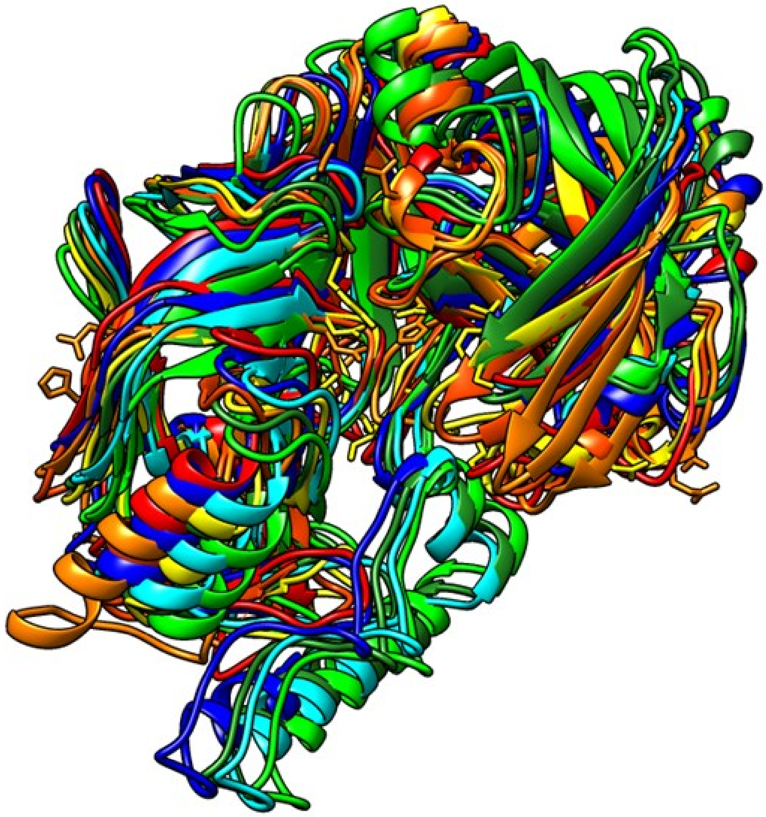

Figure S3. Superimposed image of Plasmepsins I (PDB: 3QRV; red), II (PDB:1LF4, orange red), III (PDB: 3FNS, orange), IV (1LS5, green), VI (dark green), VII (cyan), IX (sea green) and X (blue).

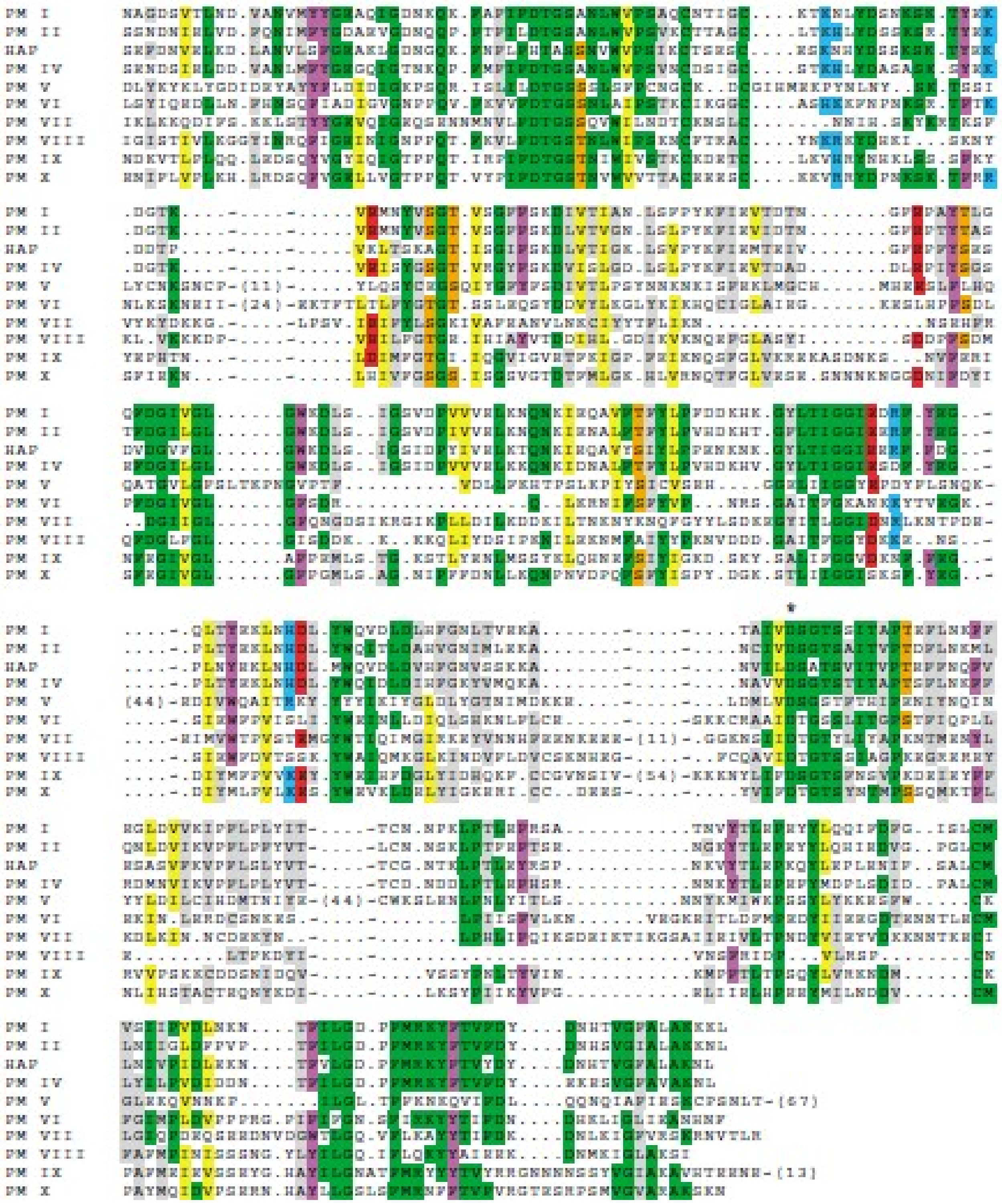

Figure S4. Sequence alignment of Plasmepsin I-X from *Plasmodium falciparum.*

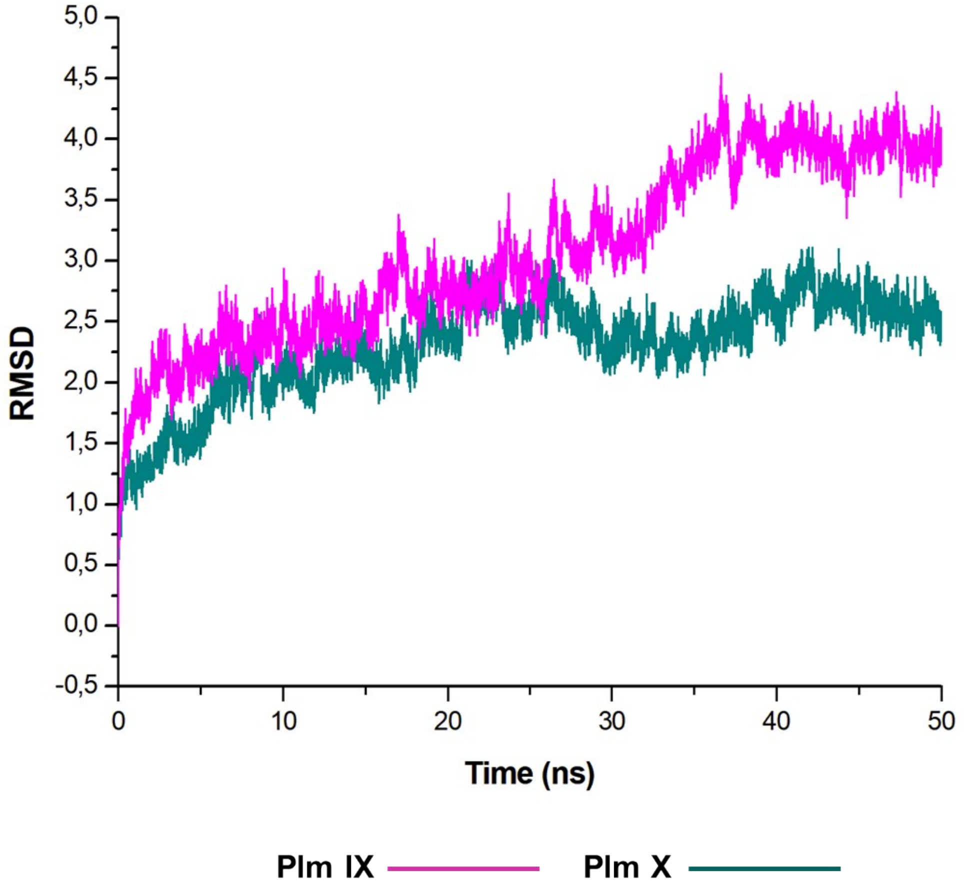

Figure S5. RMSD plot of plasmepsin IX and X, displaying system stability after 30ns.

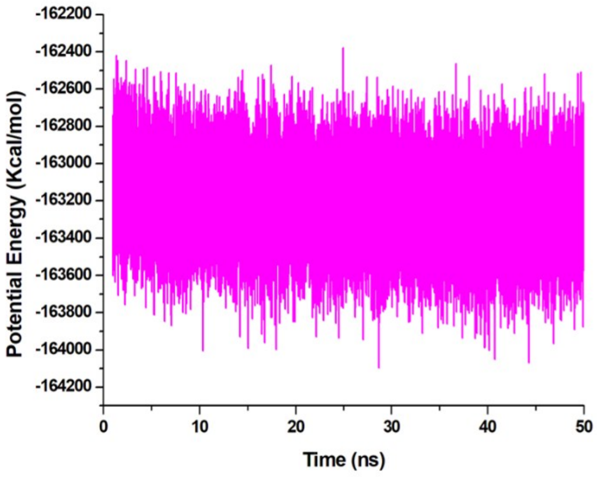

PE= -163213,16136

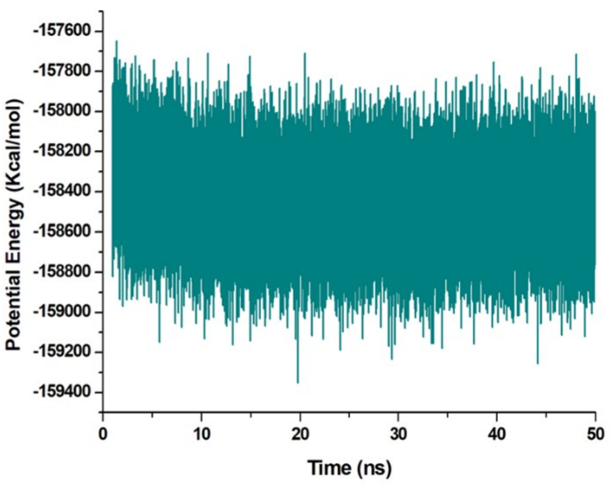

PE= -158461,37335

Figure S6. Potential energy (PE) plots of Plm IX (left) and Plm X (right) with interaction cutoff of 12 Å. Mean values are presented below the respective plots.
